# Characterization of Ixophilin, A Thrombin Inhibitor from the Gut of *Ixodes scapularis*

**DOI:** 10.1371/journal.pone.0068012

**Published:** 2013-07-09

**Authors:** Sukanya Narasimhan, Oriana Perez, Sara Mootien, Kathleen DePonte, Raymond A. Koski, Erol Fikrig, Michel Ledizet

**Affiliations:** 1 Section of Infectious Diseases, Department of Internal Medicine, Yale University School of Medicine, New Haven, Connecticut, United States of America; 2 L2 Diagnostics, New Haven, Connecticut, United States of America; 3 The Howard Hughes Medical Institute, Chevy Chase, Maryland, United States of America; The University of Texas at San Antonio, United States of America

## Abstract

*Ixodes scapularis*, the black-legged tick, vectors several human pathogens including *Borrelia burgdorferi,* the agent of Lyme disease in North America. Pathogen transmission to the vertebrate host occurs when infected ticks feed on the mammalian host to obtain a blood meal. Efforts to understand how the tick confronts host hemostatic mechanisms and imbibes a fluid blood meal have largely focused on the anticoagulation strategies of tick saliva. The blood meal that enters the tick gut remains in a fluid state for several days during the process of feeding, and the role of the tick gut in maintaining the blood-meal fluid is not understood. We now demonstrate that the tick gut produces a potent inhibitor of thrombin, a key enzyme in the mammalian coagulation cascade. Chromatographic fractionation of engorged tick gut proteins identified one predominant thrombin inhibitory activity associated with an approximately 18 kDa protein, henceforth referred to as Ixophilin. The *ixophilin* gene was preferentially transcribed in the guts of feeding nymphs. Expression began after 24 hours of feeding, coincident with the flow of host blood into the tick gut. Immunity against Ixophilin delayed tick feeding, and decreased feeding efficiency significantly. Surprisingly, immunity against Ixophilin resulted in increased *Borrelia burgdorferi* transmission to the host, possibly due to delayed feeding and increased transmission opportunity. These observations illuminate the potential drawbacks of targeting individual tick proteins in a functional suite. They also underscore the need to identify the “anticoagulome” of the tick gut, and to prioritize a critical subset of anticoagulants that could be targeted to efficiently thwart tick feeding, and block pathogen transmission to the vertebrate host.

## Introduction

*Ixodes scapularis* (*I. scapularis*) ticks transmit bacterial and protozoan pathogens, including *Anaplasma phagocytophilum* (the agent of human granulocytic anaplasmosis), *Borrelia burgdorferi* (the agent of Lyme disease) and *Babesia microti* (the agent of babesiosis), representing some of the major vector-borne infectious diseases in Central and Northeastern USA [1]. There remains an unmet need for effective vaccines against the diseases transmitted by *I. scapularis* ticks. Tick-based vaccine molecules that can block the transmission of multiple pathogens are desired, and would have an advantage over pathogen-based vaccines that target individual pathogens. Since tick feeding is intimately intertwined with pathogen transmission and acquisition, research efforts have focused on identifying tick molecules critical for tick feeding [2], [3]. The emphasis has been on tick salivary proteins that suppress and modulate host defense and haemostatic mechanisms, and impair the ability of the host to thwart tick feeding [2], [4]. However, the functional redundancy and structural paralogy inherent in the *I. scapularis* salivary gland transcriptome, and proteome [5] has confounded the development of viable salivary vaccine targets to effectively block tick feeding.

Ixodid ticks feed for 4–10 days, and blood in the gut is maintained in a fluid state throughout the process of repletion, and up to 24–48 h beyond repletion. The anticoagulation mechanisms in the gut have not been addressed at the molecular level. Ticks alternately deposit saliva and suck blood at the tick bite site [6]. It is therefore presumed that tick salivary anticoagulants deposited into the tick bite site are taken up along with the blood, and function both at the vector-host interface and in the tick gut to keep the blood fluid. We now present data to show that the tick gut is not a passive bystander, and that it plays an active role in thwarting host coagulation. We show that the tick gut expresses a thrombin inhibitor, Ixophilin, during tick feeding. These findings open up a new avenue of research, hitherto ignored, that can increase our understanding of tick feeding strategies, and provide novel targets for interrupting tick feeding and pathogen transmission.

## Materials and Methods

### Ethics Statement

Animals utilized in this study were housed and handled under the Guide for the Care and Use of Laboratory Animals of the National Institutes of Health. The animal experimental protocol was approved by the Yale University’s Institutional Animal Care & Use Committee (Protocol Number: 2012–07941). All animal infection experiments were performed in a Bio-safety Level 2 animal facility, according to the regulations of Yale University.

### Mice and Ticks

4–6 week old female C3H/HeN mice were purchased from NIH/NCI and all animal experiments were performed according to protocols approved by the Institutional Animal Care and Use Committee at the Yale University School of Medicine. *I. scapularis* nymphs and larvae were obtained from a tick colony at the Connecticut Agricultural Experiment Station in New Haven CT, USA. Tick rearing and maintenance was conducted in an incubator at 23°C with 85% relative humidity and a 14/10 h light/dark photo period regimen.

To generate *Borrelia burgdorferi*-infected nymphs, a low-passage-number clonal isolate of *B. burgdorferi* N40 that is infectious to mice [7] was used to inoculate C3H/HeN mice. Approximately, 100 µl of 1×10^5^ N40 spirochetes/ml was injected subcutaneously. Skin punch biopsies were collected from each mouse 2 weeks after inoculation and DNA isolated using the DNeasy kit (QIAGEN, Valencia, CA) and tested by quantitative PCR for the presence of spirochetes as described below. *I. scapularis* larvae (∼100/mice) were placed on each *B. burgdorferi*-infected C3H/HeN mice and fed-larvae molted to generate *B. burgdorferi*-infected nymphs. At least 15–20 unfed nymphs were dissected and guts processed for DNA extraction as described above for skin punch biopsies, and DNA tested by quantitative PCR for the presence of spirochetes as described below. Batches of nymphs that demonstrated at least 95% infection were utilized in transmission experiments.

### Preparation of Extracts

Salivary glands and midguts were dissected from engorged adult and nymphal *I. scapularis* fed to repletion on rabbits (New Zealand white) and mice (C3H/HeN). Each pair of adult salivary glands and each midgut were rinsed in PBS and then homogenized in a volume of approximately 35 µl of PBS. Engorged nymphal salivary glands were dissected and suspended in pools of 2 pairs of salivary glands and 2 guts in 35 µl of PBS. The extract was clarified by centrifugation at 14,000×*g*.

### Thrombin and Factor Xa Inhibition Assays

Purified human factor Xa (Enzyme Research Laboratories) was incubated with a colorimetric substrate (Bachem, L2115) at 25°C in the presence of varying amounts of tick extract. The final concentrations of enzyme and substrate were 312 pM and 312 µM respectively. The optical density at 405 nm was read every 15 seconds for five minutes and the rate of the reaction was determined. Purified human thrombin (Enzyme Research Laboratories) was incubated with a colorimetric substrate (Bachem L1490) at 25°C in the presence of varying amounts of tick extract. The final concentrations of enzyme and substrate were 624 pM and 312 µM respectively. The optical density at 405 nm was measured every fifteen seconds for five minutes and the rate of the reaction was determined.

The ability of protein samples to inhibit thrombin or factor Xa was assessed by adding up to ten microliters of sample in PBS to the reaction mix described above. The percent inhibition was calculated by comparing the reaction rate to the rate seen with enzyme alone.

### Chromatographic Procedures

Adult *I. scapularis* females were fed on New Zealand white rabbits as described above. The gut extract was prepared as described above. Anion exchange chromatography was performed at room temperature on a 5 ml column packed with Sepharose Q XL (GE Healthcare). Proteins were eluted with a 0 to 1 M NaCl gradient in 20 mM Tris HCl pH 8.0. Size exclusion chromatography was performed on a Superdex 200 column (GE Healthcare). The running buffer consisted of 19.6 mM KH_2_PO_4_, 30.4 mM Na_2_HPO_4_, 150 mM NaCl, pH 7.0. A one-milliliter thrombin affinity chromatography column was prepared by coupling 0.5 mg of bovine thrombin (Sigma, repurified by size exclusion chromatography) to NHS-activated Sepharose (GE Healthcare) according to the manufacturer’s instructions. Proteins to be analyzed were applied to the column in PBS. Bound proteins were eluted with 0.1 M Glycine pH 2.7 and immediately brought to neutral pH by addition of 0.5 M Tris HCl pH 9.0.

### Protein Identification

Protein identification was performed by the W. M. Keck Foundation Biotechnology Resource Laboratory at Yale University. Briefly, proteins in SDS gel slices were digested to completion with trypsin. LC MS/MS analysis of the resulting peptides was performed with either a Waters Q-tof Ultima or an LTQ Orbitrap mass spectrometer. All MS/MS spectra were searched using the automated Mascot algorithm against the NCBI nr database or against the iscapularis_preliminary_PEPTIDES_VB-IscaW1.0.5.1.fasta database. This last database contains preliminary data derived from the *I. scapularis* genome sequencing project and was current at the time of analysis.

### Tick RNA Isolation and Quantitative RT-PCR

*About* 100–200* I. scapularis* larvae were allowed to feed to repletion on each C3H/HeN mice as described earlier. Twenty five to thirty nymphal ticks were allowed to feed for 24 hours, or to repletion on experimental and control C3H/HeN mice and 10–15 female adults were allowed to feed to repletion on the ears of New Zealand white rabbit ears as described earlier [8]. Unfed and fed nymphs and adults were dissected and salivary glands and midguts were pooled in groups of 5 nymphs or individual adults, suspended in 200 µl of Trizol (Invitrogen, CA), homogenized and RNA was extracted as described earlier [9]. The same procedure was performed with unfed nymphs and adults. Unfed and fed larvae were pooled in groups of 5, ground dry under liquid nitrogen and suspended in 200 µl of Trizol and RNA extracted as described above. cDNA was synthesized using the iScript RT-PCR kit (Biorad, CA) and analyzed by quantitative PCR for the expression of tick actin and *ixophilin,* using the iQ Syber Green Supermix (Biorad, CA) on a MJ cycler (MJ Research, CA) using tick actin primers as described earlier [10] and *ixophilin* primers IxRT F and R described in Table 1.

**Table 1:** Primers utilized in this study.

Primer name	Forward primer sequence	Reverse primer sequence	Expected amplicon size (bp)
*IxA and B*	ATGAAGGCTGTCATC	GGGCTGGCAGACGAAC	427
*IxA and C*	ATGAAGGCTGTCATC	GTGCCCCTCAGAGAC	1472
*IxDESF and R*	CCATGGCAGAGGAATGCCATC	CTCGAGAGGGGGCTGGCAGACGAAC	395
*IxRTF and R*	CCCTCGCTTCTTCTACTTCAACG	CGCTGTAGAGGAAGGTTTCG	263

To obtain the full-length ixophilin, cDNA was prepared as described above from engorged nymphal guts and used as template to amplify the full-length ixophilin transcript. Primer sets IxA and B; or primer sets Ix A and C (Table 1) were used in conjunction with Platinum Taq High Fidelity polymerase (Invitrogen, CA) at an annealing temperature gradient ranging from 48 to 62°C.

### Production of Recombinant Ixophilin

Amino acid residues 16–142 of mature Ixophilin were cloned into the *Nco*I and *Xho*I restriction sites of the *Drosophila* expression vector pMT/Bip/V5-HisA plasmid (Invitrogen, CA), using the primers IxDESF and IxDESR (Table 1). Positive clones were sequenced to confirm their integrity and were transfected into *Drosophila melanogaster* S2 cells. Stable transfectants were generated as described earlier [11]. Protein expression was induced with copper sulfate as described by the manufacturer (Invitrogen, CA) and rIxophilin purified from the supernatant using Ni-NTA Superflow column chromatography (Qiagen, CA), eluted with 200 mM imidazole. Fractions containing rIxophilin as assessed by reactivity with anti-V5 antibody on a western blot (Invitrogen, CA) were pooled and concentrated using a centrifugal concentrator (Molecular weight cutoff 3 kDa, Millipore, Bedford, MA) and dialyzed against PBS. Purity of rIxophilin was assessed by Coomassie blue staining after electrophoresis on a SDS 4–20% polyacrylamide gel. Presence of glycosylation was assessed using the Glycoprotein Detection kit (Sigma-Aldrich, MO). Protein concentration was determined by Pierce BCA protein assay kit (Thermo Fisher Scientific inc., IL).

### Immunoblotting Analysis to Assess Expression, Reactivity and Molecular Weight of Native Ixophilin

Pools of 5 fed nymphal midgut extracts or 10 salivary gland extracts were electrophoresed on a SDS 4–20% polyacrylamide gel and transferred to nitrocellulose membranes. The membranes were blocked with PBS containing 5% milk powder, 0.05% Tween 20, and the immunoblots were probed with a 1∶250 dilution of mouse anti-rIxophilin serum. Immunoreactive protein bands were visualized using horseradish peroxidase conjugated goat anti-rabbit secondary antibodies (Sigma-Aldrich, MO) and the enhanced chemiluminescence Western Blotting Detection System (GE Healthcare, NJ).

### Immunization of Mice with rIxophilin and Challenge with Clean or *B. burgdorferi*-infected Nymphs

Mice were immunized subcutaneously immunized with 10 µg of rIxophilin emulsified with complete Freund’s adjuvant. Two subsequent booster immunizations of 10 µg each of rIxophilin were given emulsified in incomplete Freund’s Adjuvant at 3-week intervals. Control mice were similarly immunized with ovalbumin. Two weeks after the last immunization, sera were obtained by retro-orbital bleeding, and reactivity of sera with native and rIxophilin tested by western blot analysis as described below. Mice were then infested with 10–12 pathogen-free (clean) *I. scapularis* nymphs/mouse, and mice individually placed in metabolic cages on a metal rack separated from about 100 ml of water to contain repleted ticks. Upon repletion, nymphal ticks detached from the murine host and fell into the water in the bottom of the cage. The animals were monitored twice everyday, once at 10 A.M and once at 3 P.M, and repleted nymphs collected from each cage. Time to repletion, and engorgement weights of nymphs were recorded.

In a separate experiment, five mice immunized with ovalbumin or rIxophilin as described above, were each challenged with 4–5 *B. burgdorferi*-infected nymphs that were allowed to feed to repletion as described above. Nymphal salivary glands and guts were dissected immediately after repletion and processed for DNA extraction as described below. Mice were sacrificed 21 days post tick detachment and heart, bladder and skin aseptically removed as described earlier [12] and processed for DNA extraction as described below.

### Tick and Mouse DNA Extraction

DNA was extracted from pools of 2–3 replete nymphal salivary glands, guts and from mice tissues using the Genomic DNA Extraction Kit according to the manufacturer’s instructions (Qiagen, Valencia, CA). DNA was analyzed by quantitative PCR using the iQ Syber Green Supermix (Biorad, CA) on a MJ cycler (MJ Research, CA) for levels of *B. burgdorferi flaB* gene as a measure of *B. burgdorferi* burden using primers described earlier [12], and data normalized to tick actin or mouse actin as described earlier [12].

### Statistical Analysis

In tick feeding rate, engorgement, and quantitative PCR experiments, the significance of the difference between the mean values of control and experimental groups was analysed using the non-parametric 2-tailed Man-Whitney test with Prism 5.0 software (GraphPad Software Inc, LaJolla, CA). *P*≤0.05 was considered statistically significant.

## Results

### A Thrombin Inhibitory Activity in the Guts of Fed Adult *Ixodes scapularis* Ticks

**Figure 1:**
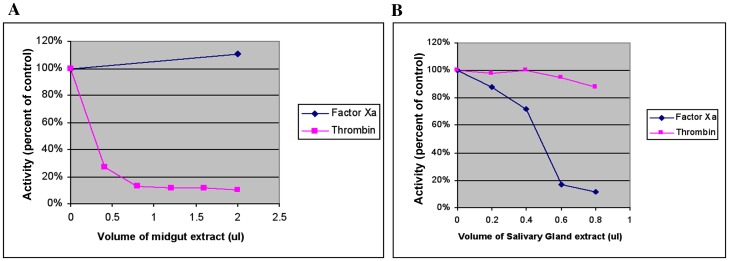
*Ixodes scapularis* gut extracts inhibit thrombin. **A**. Inhibition of factor Xa (solid diamonds) and thrombin (solid squares) by an extract from adult tick guts. Each gut was homogenized in approximately 30 µl of PBS. **B**. Inhibition of factor Xa (solid diamonds) and thrombin (solid squares) by an extract from adult tick salivary glands. Each pair of salivary glands was homogenized in approximately 30 µl of PBS.

Adult *I. scapularis* feed for 6–8 days and imbibe as much as 100 times their body weight of host blood [13]. Dissection of the replete tick gut releases fluid blood suggesting that the blood remains fluid during the process of feeding and for some 1–2 days after the feeding. We assessed if the adult guts actively prevented the coagulation of blood or if salivary anticoagulants spit into the feeding lesion are sucked into the gut and utilized for anticoagulation. Extracts from guts and salivary glands dissected from engorged adult *I. scapularis* were assayed for the ability to inhibit thrombin and factor Xa- two key enzymes in the intrinsic coagulation pathway [14]. The results showed that the adult *I. scapularis* gut extracts predominantly inhibited the activity of thrombin (Fig. 1A), with little or no activity against factor Xa. The engorged salivary gland extracts, in contrast, predominantly inhibited factor Xa with little or no activity against thrombin (Fig. 1B).

### Partial Purification of the Adult *I. scapularis* Midgut Thrombin Inhibitor

An extract from ten adult *I. scapularis* guts was fractionated by anion exchange chromatography. Fractions containing thrombin inhibitory activity were then subjected to thrombin-affinity chromatography as described in Materials and Methods. Proteins retained on the column were eluted, concentrated, and separated by SDS-PAGE. Four bands were visible, with apparent molecular weights of 14, 16, 18, and 38 kDa. Proteins present in these bands were identified by tryptic digestion and LC-MS/MS. Among the proteins detected was the product encoded by the putative *I. scapularis* gene ISCW003862. A BLAST [15] analysis of the putative gene product of ISCW003862 showed homology with Hemalin and Boophilin, two thrombin inhibitors isolated from *Haemaphysalis* and *Boophilus* ticks [16], [17], respectively.

**Figure 2:**
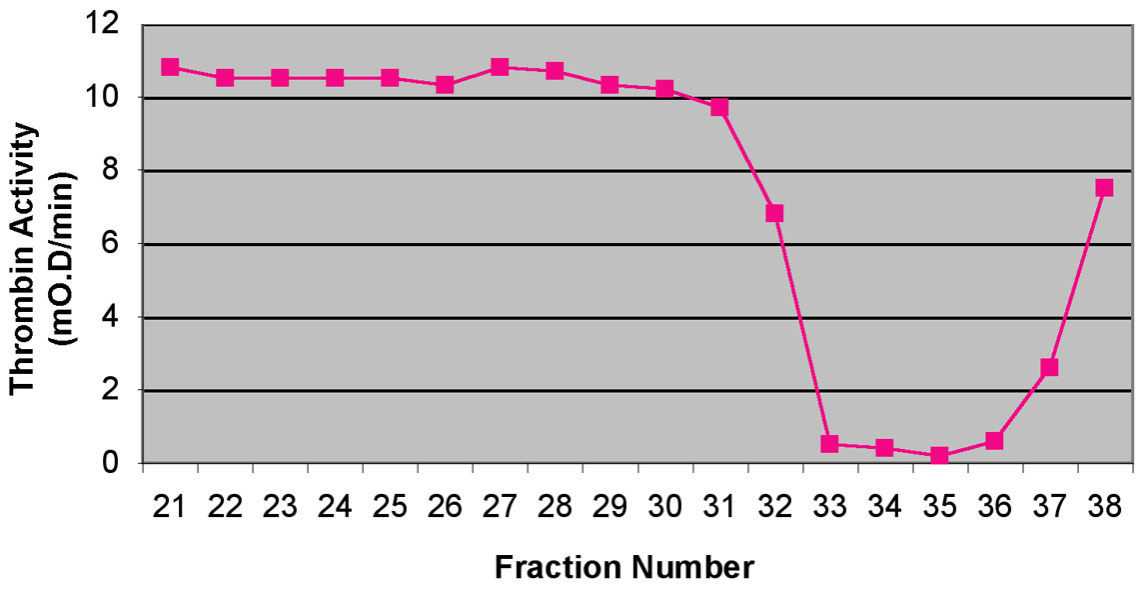
Thrombin inhibitory activity in size exclusion chromatography fractions. Anion-exchange fractionate proteins were fractionated on a Superdex S200 column as described in Materials and Methods. Each enzymatic assay included 7.5 µl of the indicated column fraction.

In a separate approach, an extract prepared from thirty engorged adult *I. scapularis* guts was fractionated by anion exchange followed by size exclusion chromatography. All fractions were assayed for thrombin and Factor Xa inhibitory activity as described in Materials and Methods. The thrombin inhibitory activity eluted in a single peak (Fig. 2). There was no Factor Xa inhibitory activity associated with these fractions. Fractions with maximal thrombin inhibitory activity were pooled and analyzed by SDS PAGE. Predominant bands were dissected and proteins contained therein identified by tryptic digestion and LC MS/MS. Again, we noted the presence of a protein derived from the putative gene ISCW003862 in a band with an apparent molecular weight of 18 kDa. On the basis of its homology with Boophilin, we will use the name Ixophilin when referring to the product of the *Ixodes scapularis* putative gene ISCW003862.

### Full-length *ixophilin* mRNA Encodes a 15 kDa Protein

**Figure 3:**
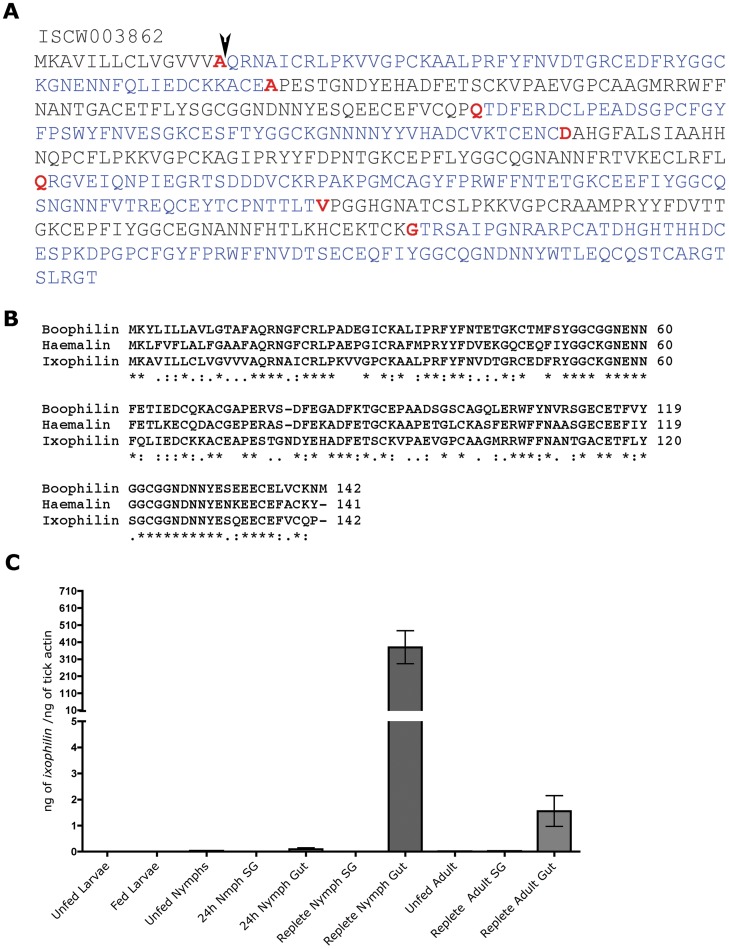
Ixophilin shows homology with thrombin inhibitors, and is preferentially expressed in the guts of nymphal and adult *Ixodes scapularis*. **A.** Annotated sequence of protein encoded by ISCW003862 (Ixophilin). Alternating colors indicate exons, aminoacids underlined in red indicate the end of each exon. Arrow-head indicates the signal cleavage site between Alanine and Glutamine. **B.** ClustalW alignment of Ixophilin, Boophilin [17] and Hemalin [16]. Asterisks (*) indicate identity between all three sequences; colon (:) indicates conservation between groups of strongly similar properties; period (.) indicates conservation between groups of weakly similar properties - scoring = /<0.5 in the Gonnet PAM 250 matrix. **C.** Quantitative RT-PCR analysis of *ixophilin* expression in unfed, fed larvae, unfed, 24 h fed, and replete nymph salivary glands (SG) and guts (Gut); and in unfed and replete adult salivary glands (SG) and guts (Gut). Error bars represent mean±SEM of 4 replicates in pools of 5 salivary glands and guts.

The annotation of ISCW003862 predicts that the gene encompasses 8 exons and encodes a 54.4 kDa protein (Fig. 3A), considerably longer than the ∼20 kDa thrombin inhibitors Boophilin and Hemalin [16], [17]. The genome annotation also predicts a serine protease inhibitor function for the ISCW003862 encoded protein. The annotated gene structure of ISCW003862 shows that the splicing of 8 exons defined by canonical intron-exon splice junctions generates the transcript ISCW003862RA. However, our attempts to amplify this 1476 bp transcript of ISCW003862RA using primers IxA and IxC complementary to Exon1 and 8 (Table 1) were unsuccessful. Based on the homology with the thrombin inhibitors identified from the guts of *H. longicornis* [16] and *B. microplus* [17] (Fig. 3B), we hypothesized that ISCW003862 might be composed solely of the first 3 exons. Splicing of these exons would generate a 429 bp transcript encoding a 143 amino acid long protein, congruent with the full-length sequences of Boophilin and Hemalin (Fig. 3B). Primers IxA and IxB based on this possible 3-exon structure (Table 1) provided an expected amplicon encoding the putative full-length transcript of ISCW003862, and henceforth referred to as *ixophilin*, from adult tick gut cDNA. The three-exon structure of *ixophilin* is further confirmed by the sequencing results of native Ixophilin. The peptides recovered from fractions with thrombin inhibitory activity (FYFNVDTGRCEDFR, GNENNFQLIEDCK(K), ACEAPESTGNDYEHADFETSCK, and VPAEVGPCAAGMR), are all encoded by the first three exons of ISCW003862. We did not detect any peptide derived from exons 4 through 8 of ISCW003862. The full-length sequence of Ixophilin was analyzed using the Signal P3.0 software (www.cbs.dtu.dk/services/SignalP/) and a signal peptide indicative of a secretory protein was predicted with a significant probability score of 0.998 and signal peptide cleavage site predicted between amino acid residues 15 and 16. Analysis of the protein using the ExPasy proteomics software suite (www.expasy.org/proteomics) revealed one potential O-glycosylation site on the tyrosine residue at position 43 (Fig. 3A).

### Ixophilin is Expressed in all Life Stages of *I. scapularis* and is Induced upon Feeding

Larval, nymphal and adult stages of *I. scapularis* were assessed for expression of *ixophilin* by quantitative RT-PCR. cDNA generated from unfed and fed whole larval RNA and from unfed and fed nymphal and adult guts and salivary glands was used as template to PCR amplify *ixophilin* transcripts. *Ixophilin* expression was induced upon feeding in all nymphal and adult stages (Fig. 3C), and preferentially expressed in the guts of nymphs and adults (Fig. 3C). These observations confirm that the thrombin inhibitory activity does not originate from the salivary proteome. Further, expression levels of *ixophilin* were highest in the nymphal stage compared to that in the adult stage (Fig. 3C). Expression of *ixophilin* in the unfed larval stage was 100,000-fold lower than in the nymphal and adult stage, and was not increased upon feeding (Fig. 3C).

### Recombinant Ixophilin Inhibits Thrombin Activity

**Figure 4:**
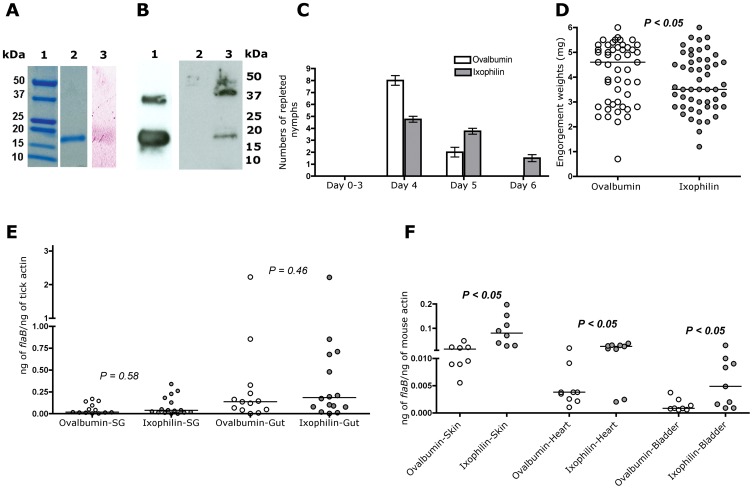
Immunization with rIxophilin delays time to repletion and enhances *Borrelia burgdorferi* burden in the murine host. **A.** Electrophoresis of rIxophilin on a 4–20% SDS PAGE. Lane 1, Precision Plus protein standards (BioRad); Lane 2, Coomassie Blue stain of rIxophilin; Lane 3, Glycoprotein stain of rIxophilin. **B.** Western blot probed with serum from a mouse immunized with rIxophilin. The proteins analyzed were: rIxophilin (lane 1); salivary gland extract prepared from 10 fed nymphs (lane 2); and gut extract prepared from 5 fed nymphs. **C.** 10–12 pathogen-free nymphs were fed on each of five ovalbumin or rIxophilin-immunized mice, and time to repletion in each group recorded. Error bars represent mean±SEM. **D.** Engorgement weights of nymphs fed on ovalbumin or rIxophilin-immunized mice. Each data point indicates one nymph. **E.** Quantitative PCR assessment of *flaB* levels as a measure of *B. burgdorferi* burden in guts (Gut) and salivary glands (SG) of nymphs fed to repletion on each ovalbumin or rIxophilin-immunized mice. Each data point represents a pool of 2 guts or salivary glands. **F.** Quantitative PCR assessment of *flaB*levels as a measure of *B. burgdorferi* burden in skin, bladder and heart tissues of ovalbumin or rIxophilin-immunized mice 21 days post-tick detachment. Each data point represents tissue from one mouse. E and F represent a composite of 2 replicate experiments. Horizontal bars represent the median, and mean values significantly different in a two-tailed non-parametric t-test (P<0.05) indicated.

To further confirm that Ixophilin encoded a thrombin inhibitor, recombinant mature Ixophilin (amino acids 16 through 143) was expressed in *Drosophila* cells using the Drosophila Expression System (Invitrogen) as described in Materials and Methods. Recombinant Ixophilin (rIxophilin) had an apparent mass of 18 kDa and was purified to near homogeneity as seen by Coomassie blue staining (Fig. 4A). Glycoprotein staining suggested the presence of moderate amounts of glycosylation on the recombinant protein (Fig. 4A). The purified protein was assayed for its ability to inhibit thrombin as described in Materials and Methods. Addition of 1–5 µg of rIxophilin to standard assays resulted in a 10–20% decrease in thrombin activity. Addition of up to 10 µg of unrelated native and recombinant proteins did not cause any change in thrombin activity, confirming the specificity of the action of rIxophilin.

### Immunity against Ixophilin Decreases Feeding Efficiency of Nymphs, but Increases *Borrelia burgdorferi* Transmission to Mice

Five C3H/HeN mice were immunized with rIxophilin according to a standard immunization regimen. In parallel, five control mice were immunized with ovalbumin. Antibodies from mice immunized with rIxophilin generated in the *Drosophila* expression system (Fig. 4B, lane 1) reacted with a ∼18 kDa protein corresponding to Ixophilin protein in the fed nymphal gut (Fig. 4B, lane 3). We also observed a cross-reacting ∼36 kDa protein in the purified rIxophilin preparation (Fig. 4B, lane 1), and a ∼ 38 kDa protein in the gut extracts (Fig. 4B, lane 3). Since this cross-reacting band was observed in the purified rIxophilin preparation, we reason that this might represent a dimer of Ixophilin. rIxophilin and ovalbumin-immunized mice were each challenged with 10–12 pathogen-free *I. scapularis* nymphs that were allowed to feed to repletion. Nymphs on Ixophilin-immunized mice took longer to feed to repletion (Fig. 4C) and reached significantly decreased engorgement weights compared to nymphs that fed on ovalbumin-immunized mice (Fig. 4D). We therefore examined whether transmission of pathogens by *I. scapularis* nymphs might be influenced by the decreased feeding efficiency. C3H/HeN mice immunized with rIxophilin or ovalbumin as described above were each challenged with 4–5 *B. burgdorferi*-infected nymphs that were allowed to feed to repletion. The *Borrelia* burden in the guts and salivary glands of replete nymphs was not significantly influenced by the decreased feeding efficiency (Fig. 4E). However, contrary to our expectation, *Borrelia* burden in the vertebrate host was significantly increased in the skin, heart and bladder of rIxophilin-immunized mice (Fig. 4F).

## Discussion

Tick anticoagulation strategies are central to successful feeding and it is now well documented that the tick utilizes a multi-pronged strategy to thwart host hemostasis [14], [18], [19]. However, the functional redundancy of the salivary “anticoagulome” poses a major bottleneck in efforts to develop vaccines targeting specific salivary anticoagulants [4], [20], [21], [22]. We now shift the focus from the tick saliva to the tick gut and draw attention to the role of the tick gut in anticoagulation and reveal a new critical aspect in tick feeding. Ixodid ticks feed for 4–10 days and imbibe as much as 100 times their body weight of blood meal during engorgement [13]. The tick gut serves as a storage organ for fluid blood conducive for both receptor-mediated and fluid-phase endocytosis of the blood meal by the gut digestive cells [23]. The blood in the gut is maintained in a fluid state throughout the process of repletion and up to 24–48 hours beyond repletion. Ticks alternately deposit saliva into and suck blood from the tick bite site [6], and it was presumed that salivary immune modulators and anticoagulants deposited into the bite site, and absorbed into the gut along with the bloodmeal might provide essential functions both at the bite site and in the gut. However, a recent analysis of the gut transcriptome of *Dermacentor variabilis* by Anderson *et al* [24] showed that about 6% of the sequenced transcripts encoded secreted proteins with putative antioxidant, anticoagulant and antimicrobial functions distinct from that observed in salivary glands. Consistent with this emerging active role for the tick gut in feeding, several reports demonstrate the expression of gut-specific anticoagulants in several Ixodid ticks [16], [17], [25], [26].

We now show that the *I. scapularis* adult gut elaborates an anticoagulant activity predominantly inhibiting the thrombin step of the intrinsic pathway of host coagulation. The lack of activity against factor Xa (Fig. 1A) is in clear contrast to the predominant factor Xa inhibitory activity observed in tick saliva [27]. We observed minimal thrombin inhibitory activity in adult saliva collected from engorged adult ticks (Fig. 1). Recently, a 23 kDa protein (P23) was identified from a nymphal salivary gland yeast display library [11] that appears to inhibit the formation of thrombin by targeting the activated factor Xa complex that precedes thrombin formation. A study by Chmelar *et al* [28] has also shown that *Ixodes ricinus* salivary protein IRS-2 inhibits thrombin activity, albeit, at very high concentrations.

We partially purified the thrombin inhibitory activity from adult tick guts by liquid chromatography (Fig. 2). LC-MS/MS of the peptides in the active peak revealed the presence of a protein derived from the ISCW003862 locus. We named this protein Ixophilin on the basis of its strong homology with the thrombin inhibitors Boophilin [17] and Hemalin [16] from *Boophilus microplus* and *Haemaphysalis longicornis* respectively. ClustalW2 (www.ebi.ac.uk/Tools/msa/clustalw2) alignment of the full-length sequences of Ixophilin, Boophilin and Hemalin revealed 50% identity and 27% similarity over the 142 amino-acid full-length Hemalin and Boophilin (Fig. 3B). Ixophilin showed homology with Kunitz-domain containing super family of putative serine protease inhibitors, and contained 2 Kunitz domains,-one spanning amino acid residues 21 through 75, and a second, spanning residues 95 through 140. Kunitz domains have traditionally served as a preferred scaffold for evolution of tick anticoagulants [29], [30], [31]. Taken together, our observations suggest that Ixophilin is responsible, at least in part, for the thrombin inhibitory activity found in the tick midgut. rIxophilin showed the ability to inhibit thrombin by about 10–20%. The activity of rIxophilin was much lower than the approximately 90% thrombin inhibitory activity observed in the native gut protein fractions (Fig. 2), possibly due to the absence of tick-specific post-translational modifications, or to incorrect folding of rIxophilin. Ixophilin-derived peptides were obtained from SDS-PAGE bands with apparent molecular weights of 14, 16, and 18 kDa following thrombin-affinity chromatography, suggesting that Ixophilin may be activated by proteolytic cleavage. Incubation of rIxophilin with extracts from salivary glands and from midguts failed to increase the activity of the recombinant protein (data not shown). Despite our inability to generate a potent rIxophilin, the sequence homology of Ixophilin with two other proteins (Fig. 3), Boophilin and Hemalin that have been shown conclusively to be thrombin inhibitors [16], [17], garners support for a thrombin inhibitory role for Ixophilin in the tick gut.

Temporal and spatial analysis of *ixophilin e*xpression showed that it was preferentially expressed in the adult and nymphal gut and was induced upon feeding (Fig. 3), consistent with a potential role for Ixophilin in preventing the clotting of the blood meal in the gut. *Ixophilin* expression levels were 100–200-fold higher in the nymphal gut compared to the adult gut (Fig. 3). *ixophilin* expression levels in fed and unfed larval stages were comparable, and significantly lower than that in nymphal and adult guts, suggesting that Ixophilin was possibly not the predominant anticoagulant in the larval stage (Fig. 3C). Since *ixophilin* expression was higher in the nymphal gut, we assessed the role of Ixophilin in nymphal feeding. Further, expression levels of ixophilin were significantly increased in repleting ticks (Fig. 3), coincident with the rapid phase of tick feeding [32], and of rapid intracellular blood meal digestion [33]. The activity of Ixophilin might, in part, be instrumental in keeping the blood stored in the tick gut in a fluid state, and thus allow nutrient uptake by pinocytosis-an important aspect of blood meal digestion in ticks [33].

Immunity against Ixophilin significantly delayed feeding time, and decreased engorgement weights (Fig. 4C–D), but did not abolish feeding. Interestingly, rIxophilin-immunized mice challenged with *B. burgdorferi*-infected nymphs, showed increased *Borrelia* burden in the murine host (Fig. 4F), although there was no impact on *Borrelia* burden in the nymphal guts and salivary glands (Fig. 4E). Presumably, the longer feeding period might have increased the window of time for pathogen transmission to occur. The tick gut may encode more than one anticoagulant that may compensate for loss of Ixophilin function, and allow feeding to proceed, and emphasizes the need to simultaneously target all predominant members of the anticoagulome to efficiently block tick feeding.

Our observations suggest stage-specific and tissue-specific expression of Ixophilin, and underscore a critical role for the tick gut in keeping the blood meal fluid. Unlike the salivary anticoagulants, the gut-specific anticoagulants are not directly exposed to host immune pressure, and may not have evolved to circumvent host immune responses [34], [35]. The gut “anticoagulome” might therefore be a tractable subset, and could be targeted to disrupt tick feeding, and consequent pathogen transmission.
